# Super Magnetic Niosomal Nanocarrier as a New Approach for Treatment of Breast Cancer: A Case Study on SK-BR-3 and MDA-MB-231 Cell Lines

**DOI:** 10.3390/ijms22157948

**Published:** 2021-07-26

**Authors:** Elham Jamshidifar, Faten Eshrati Yeganeh, Mona Shayan, Mohammad Tavakkoli Yaraki, Mahsa Bourbour, Ali Moammeri, Iman Akbarzadeh, Hassan Noorbazargan, Nikoo Hossein-Khannazer

**Affiliations:** 1 Department of Pharmaceutical Nanotechnology, Faculty of Pharmacy, Tehran University of Medical Sciences, Tehran 1417935840, Iran; elham.jamshidifar1373@gmail.com; 2 Department of Chemistry, Science and Research Branch, Islamic Azad University, Tehran 1417935840, Iran; 3 Department of Biology, Mashhad Branch, Islamic Azad University, Mashhad 1696700, Iran; monashayan771@yahoo.com; 4 Research and Development Department, Nanofy Technologies Pte. Ltd., Singapore 049422, Singapore; 5 Department of Biotechnology, Alzahra University, Tehran 1993891176, Iran; m.bourbour1@yahoo.com; 6 School of Chemical Engineering, College of Engineering, University of Tehran, Tehran 14174, Iran; moameriali21@gmail.com; 7 Gastroenterology and Liver Diseases Research Center, Research, Institute for Gastroenterology and Liver Diseases, Shahid Beheshti University of Medical Sciences, Tehran 6718773654, Iran; Imanakbarzadeh71@yahoo.com; 8 Department of Biotechnology, School of Advanced Technologies in Medicine, Shahid Beheshti University of Medical Sciences, Tehran 6718773654, Iran; h.noorbazargan@sbmu.ac.ir

**Keywords:** co-delivery, magnetic, niosome, silica, breast cancer, chemotherapy

## Abstract

In the present study, a magnetic niosomal nanocarrier for co-delivery of curcumin and letrozole into breast cancer cells has been designed. The magnetic NiCoFe_2_O_4_ core was coated by a thin layer of silica, followed by a niosomal structure, allowing us to load letrozole and curcumin into the silica layer and niosomal layer, respectively, and investigate their synergic effects on breast cancer cells. Furthermore, the nanocarriers demonstrated a pH-dependent release due to the niosomal structure at their outer layer, which is a promising behavior for cancer treatment. Additionally, cellular assays revealed that the nanocarriers had low cellular uptake in the case of non-tumorigenic cells (i.e., MCF-10A) and related high viability but high cellular uptake in cancer cell lines (i.e., MDA-MB-231 and SK-BR-3) and related low viability, which is evidenced in their high cytotoxicity against different breast cancer cell lines. The cytotoxicity of the letrozole/curcumin co-loaded nanocarrier is higher than that of the aqueous solutions of both drugs, indicating their enhanced cellular uptake in their encapsulated states. In particular, NiCoFe_2_O_4_@L-Silica-L@C-Niosome showed the highest cytotoxicity effects on MDA-MB-231 and SK-BR-3 breast cancer cells. The observed cytotoxicity was due to regulation of the expression levels of the studied genes in breast cancer cells, where downregulation was observed for the Bcl-2, MMP 2, MMP 9, cyclin D, and cyclin E genes while upregulation of the expression of the Bax, caspase-3, and caspase-9 genes was observed. The flow cytometry results also revealed that NiCoFe_2_O_4_@L-Silica-L@C-Niosome enhanced the apoptosis rate in both MDA-MB-231 and SK-BR-3 cells compared to the control samples. The findings of our research show the potential of designing magnetic niosomal formulations for simultaneous targeted delivery of both hydrophobic and hydrophilic drugs into cancer cells in order to enhance their synergic chemotherapeutic effects. These results could open new avenues into the future of nanomedicine and the development of theranostic agents.

## 1. Introduction

Breast cancer is one of the most common cancers in women. In 2020, the number of female breast cancer patients increased dramatically, with an estimated 2.3 million new cases, while the fatality rate of this malignant disease was 6.9% [1]. In addition to surgery and radiotherapy, the use of chemotherapy as well as targeted therapy has increased for the treatment of breast cancer, along with the availability of supportive therapies for the management of side effects [2]. To overcome the side effects of chemotherapy, a targeted drug delivery approach using highly biocompatible nanomaterials has received tremendous attention in recent years, whereby the wet synthesis approach allows for step-by-step synthesis of nanocarriers with a controllable size at a large scale and controllable surface functionality to be able to interact with the target tissue specifically [3,4,5,6,7]. Based on current evidence, nanocarriers mostly suffer from either poor solubility of anticancer drugs (i.e., hydrophilic or hydrophobic) or cytotoxicity on healthy tissue [8,9]. Therefore, a combination of nanocarriers that have potential for delivering both hydrophobic and hydrophilic molecules with nanomaterials that provides the targeted features could be a promising approach for efficient drug delivery [10,11,12].

Moreover, most anti-breast cancer drugs have a high maintenance dose with a hydrophobic nature that potentially induces low serum bioavailability, hepatic omission, and, in turn, a low level of absorption. It has been reported that poor water solubility and rapid metabolism are crucial drawbacks of various drugs in breast cancer therapy [13,14,15]. In addition, several drugs with different structures are usually administrated to achieve efficient chemotherapy [2,16].

In recent years, natural compounds have been comprehensively studied for curing diseases owing to their various characteristic activities, such as inducing tumor-suppressing autophagy and acting as antimicrobial agents. In particular, curcumin has received much attention due to its bio-functional influence through anticancer, antioxidant, and anti-inflammatory activities over twenty years [17]. One of the essential functions of curcumin is its incomparable in vitro anticancer activities, which include inhibiting proliferation, tumor invasion, and apoptosis by regulating numerous cellular signaling pathways [18,19]. It inhibits and suppresses the proliferation of cancer cells and uses its impacts by diminishing the frequency of anti-apoptotic gene products, activating caspase, and upregulating cancer-suppressive genes such as *P53* [20,21,22]. Recent investigations have declared the inhibitive and therapeutic effects of curcumin on different types of cancers, showing that it can prevent or decrease the formation or promotion of tumors. Curcumin prevents tumor invasion by alleviating the modification of matrix metalloproteases (MMPs); the cell surface adhesive molecules *AP*-1, *TNF-α*, *LOX*, and *COX*-2; chemokines and growth factors (*HER*-2 and *EGFR*), and it suppresses *N*-terminal activity and tyrosine kinase protein [23,24,25]. Curcumin acts as a prohibitor through angiogenesis in some tumors by suppressing angiogenic cytokines such as *IL*-6, *IL*-23, and *IL*-1*β* [26,27]. Due to the strong link between inflammation and cancer, the anti-inflammatory impacts of curcumin would well explain its anti-tumor effects. Curcumin also acts as a chemosensitizer that makes tumor cells more sensitive to the effects of chemotherapy, and it is used in order to tackle cancer, governed by blocking the NFκB signaling pathway and reducing overexpression of P-glycoprotein and Bcl-2 [25,28].

Investigations around letrozole also show that it has been used as a second line of therapy for advanced breast cancer. This drug has an essential role in addition to other endocrine therapies, such as tamoxifen, and improves disease-free and distant disease-free survival as well as increasing the overall objective response rate [29,30,31]. Letrozole acts as an inhibitor of aromatase and is used for breast cancer, which is hormonally positive in postmenopausal females. This drug has low water solubility, express metabolism, and a huge number of side effects. Entrapping letrozole into nanoparticle technology would enhance the duration of release and increase the sustaining capability. This process can be implemented by using biodegradable polymers [32].

Delivery of multiple drugs with a single drug nanocarrier has generated remarkable research interest for decades [14,33]. Breast cancer therapy with both letrozole and curcumin can potentially prevent xenografted endometrial tumor growth by inducing apoptosis of tumor cells. Combination of letrozole and curcumin further improves the inhibition of tumor growth [34,35]. Furthermore, in order to increase the solubility and bioavailability of curcumin and letrozole, considerable attempts have been made to encapsulate these two types of drugs in liposomes, polymeric and lipo-nanoparticles, biodegradable microspheres, cyclodextrin, and hydrogels [33,34,35,36,37]. However, targeted co-delivery of these two drugs using a highly biocompatible magnetic niosomal formulation has not been reported yet, which could further improve our insight for designing new smart nanocarriers to fight cancer.

During the current research, we designed a novel supermagnetic niosomal formulation as a potential targeted drug delivery system to deliver both hydrophilic and hydrophobic drugs into cancer cells. To achieve this, NiCoFe_2_O_4_ nanoparticles were synthesized and used as the supermagnetic core. A thin layer of silica allowed us to enhance the biocompatibility as well as the formation of the niosomal structure around the magnetic core. Letrozole and curcumin were loaded into the as-designed nanocarrier via different strategies. Different breast cell lines including both cancerous and healthy cells were used as cell models to investigate the chemotherapeutic efficacy of the as-designed nanocarriers. The results showed that our approach was highly effective and non-invasive, which led to eradicating breast cancer cells via several pathways.

## 2. Result and Discussion

### 2.1. Characterization of Nanostructures

**Figure 1:**
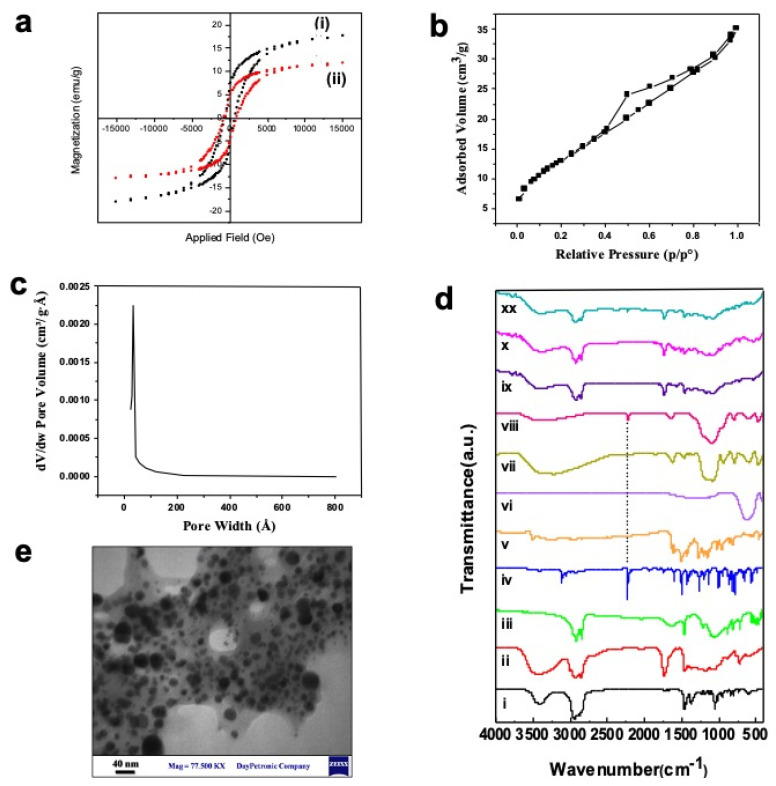
(**a**) Magnetic properties of NiCoFe_2_O_4_ nanoparticles (i) and NiCoFe_2_O_4_@Silica nanoparticles (ii); (**b**) nitrogen adsorption–desorption isotherms of the mesoporous NiCoFe_2_O_4_@Silica; (**c**) pore size distribution for NiCoFe_2_O_4_@Silica nanoparticles. (**d**) Fourier transform infrared (FTIR) spectra of (i) cholesterol, (ii) span 80, (iii) DCP, (iv) letrozole, (v) curcumin, (vi) NiCoFe_2_O_4_, (vii) NiCoFe_2_O_4_@Silica, (viii) NiCoFe_2_O_4_@L-Silica, (ix) NiCoFe_2_O_4_@L-Silica@Niosome, (x) NiCoFe_2_O_4_@L-Silica@C-Niosome, and (xx) NiCoFe_2_O_4_@L-Silica@L-Niosome; (**e**) TEM image for NiCoFe_2_O_4_@Silica@Niosome nanocarriers.

The core of the targeting drug delivery system in this work is based on using supermagnetic nanoparticles. Therefore, the supermagnetic properties of the as-synthesized NiCoFe_2_O_4_ and NiCoFe_2_O_4_@Silica nanoparticles were investigated. A vibrating sample magnetometer (VSM) was used with an applied magnetic field ranging from −15 to 15 kOe, and the typical VSM plot for each sample at room temperature is shown in Figure 1a; the results of the analysis of these curves are presented in Table S2. The super-paramagnetic properties of spinel ferrites doped with metals could be advantageous for additional control of the amount of material applied for their biological and therapy applications. In particular, NiCoFe_2_O_4_ nanoparticles possessed 17.58 emu/g in their magnetic curve. Additionally, the magnetic characteristic of NiCoFe_2_O_4_@Silica (11.71 emu/g) is slightly weaker than that of NiCoFe_2_O_4_ nanoparticles. This was expected as silica is a non-magnetic medium, which restricts the magnetic interaction between the particles, and in turn, it reduces values of saturation magnetization and the remnant field [38]. Increasing the amount of silica as the non-conductor leads to diminishing the magnetic field in NiCoFe_2_O_4_@Silica nanoparticles. However, the magnetic properties of the NiCoFe_2_O_4_@Silica nanoparticles are still considerable and efficient. Regarding the pattern of NiCoFe_2_O_4_@Silica, the ferromagnetic components are evident, which means that NiCoFe_2_O_4_@Silica does not lose its magnetic effect, as this nanoparticle has magnetic memory. This is illustrated by the S shape in Figure 1a, which has not passed from the zero center.

Since the letrozole drug molecules should be loaded into the silica shell, the porosity and surface area of NiCoFe_2_O_4_@Silica were measured. The pore size distribution (PSD) curve is shown in Figure 1c and the nitrogen adsorption–desorption isotherms of NiCoFe_2_O_4_@Silica nanoparticles are shown in Figure 1b. According to the results, the as-synthesized NiCoFe_2_O_4_@Silica nanoparticles possessed a mesopore size distribution in the range of 20.83–44.55 A°. Figure 1b shows that the adsorption isotherm at a relative pressure (P/P_0_) from 0.3 to 1.0 is IV-type, including a hysteresis loop. The existence of a noticeable hysteresis loop in the isotherm is ascribed to the presence of mesopores among the nanoparticles, which is in agreement with previous studies [39].

The Brunauer–Emmett–Teller (BET) surface area, single point total pore volume, and pore diameter of NiCoFe_2_O_4_@Silica nanoparticles were 49.07 m^2^/g, 0.0533 m^3^/g, and 43.11 Å, respectively. The pore size obtained in this study is consistent with the previously published literature, indicating successful formation of the silica shell on the surface of supermagnetic NiCoFe_2_O_4_ NPs.

The sample was further characterized using FTIR spectroscopy in the wavenumber range of 400–4000 cm^−1^. As can be seen in Figure 1d, the IR spectrum for a NiCoFe_2_O_4_@Silica nanoparticle was obtained in a spinel structure; the cations are distributed in sublattices known as tetrahedral and octahedral sites, which agrees with the literature [38]. For ferrites, the higher wavenumber band (n1) appears between 500 and 600 cm^−1^, whereas the lower wavenumber band (n2) appears between 400 and 450 cm^−1^ [40]. The bands observed around 618 cm^−1^ (n1: tetrahedral site) and 400 cm^−1^ (n2: octahedral site) correspond to stretching vibrations of Fe-O and Co-O bonds at tetrahedral sites and vibrations of oxygen perpendicular to that of tetrahedral ions, respectively [41]. The growth of silica shells around NiCoFe_2_O_4_ nanoparticles resulted in the appearance of Si-OH and Si-O-Si asymmetric vibration peaks at 790 cm^−1^ and a band around 900–1200 cm^−1^, respectively. The appearance of the C≡N bond of the letrozole molecule around 2250 cm^−1^ confirms the successful loading of letrozole molecules into the porous silica shell layer, which is in agreement with other letrozole-loaded nanocarriers [42].

The surface chemistry of the supermagnetic niosomal nanocarriers was investigated at different stages of the synthesis as well as drug loading. As can be seen in Figure 1d, the peaks at 400 and 618 cm^−1^ could be attributed to Co-O and Fe-O bonds, respectively. The growth of silica shells around NiCoFe_2_O_4_ nanoparticles results in the appearance of Si-OH and Si-O-Si peaks at 790 cm^−1^ attributed to the C≡N bond in the letrozole molecule, confirming the successful loading of letrozole molecules into the porous silica shell layer. In NiCoFe_2_O_4_@L-Silica@Niosome, the outer layer is exposed to infrared light, and therefore, the observed peaks are mostly related to the usual niosomal structure. In particular, C–O stretching, C=O stretching, C-H stretching, OH stretching, and Aliphatic C-N stretching appeared at 1125, 1747, 2800–3000, 3452, and 1000–1250 cm^−1^, respectively. Similar to a typical niosomal structure, loading drug molecules into the niosomal structure will not change the FTIR spectra of the nanocarrier, which could be due to the complete encapsulation of drug molecules inside the niosomal layer.

The changes in the hydrodynamic diameter and size distribution of the as-developed magnetic niosomal nanocarriers at different stages of formulation were monitored using the dynamic light scattering (DLS) technique. Table 1 summarizes the results of these investigations. According to the results, the average hydrodynamic diameter of bare magnetic nanoparticles increased following the formation of the silica layer and the niosomal layer. These increasing values confirm the successful formation of each of these layers on top of the magnetic nanoparticles, which is in agreement with the FTIR results. In addition, we observed that drug loading into the nanocarriers at each step also resulted in a slight increase in the average hydrodynamic diameter, which confirms the successful loading of the drugs. In the case of silica, the increase in the size of the nanocarriers after loading letrozole suggests that letrozole molecules have not only been adsorbed into the pores of the silica layer, but also they form a mono-layer at the outer surface of the silica shell. In the case of niosome, drug molecules interact with the surfactant and other components in the niosomal formulation; in turn, a slight increase in the hydrodynamic size can be observed, which is in agreement with our previous studies [16]. In addition to the DLS, the morphology of the as-designed magnetic niosomal formulation was investigated by transmission electron microscopy (TEM). As can be seen in Figure 1e, the as-synthesized nanocarriers have a spherical morphology with uniform size distribution, where the average size of the as-designed magnetic niosomal formulation is less than 35 nm.

### 2.2. Encapsulation Efficiency and Release Study of Curcumin and Letrozole

The encapsulation efficiency of letrozole and curcumin was evaluated under various conditions after adding these drugs into nanoparticles. The encapsulation efficiency of letrozole in NiCoFe_2_O_4_@Silica formulation was 61.32%. Moreover, its entrapment efficiency in NiCoFe_2_O_4_@L-Silica@Niosome reached 92.73%, while the entrapment efficiency of curcumin in NiCoFe_2_O_4_@L-Silica@C-Niosome was 81.21%.

**Figure 2:**
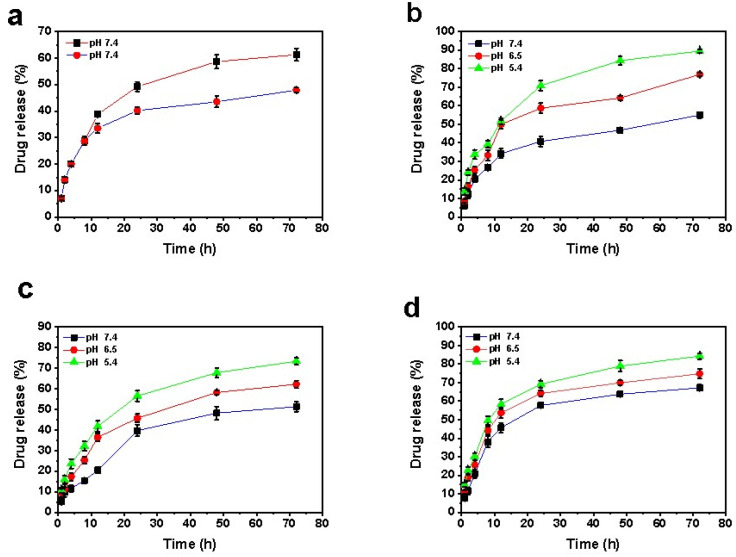
In vitro drug release profile of (**a**) letrozole from NiCoFe_2_O_4_@L-Silica (black square) and NiCoFe_2_O_4_@L-Silica@Niosome (red circle) at pH = 7.4; (**b**) letrozole from NiCoFe_2_O_4_@L-Silica@L-Niosome at different pH; (**c**) letrozole from NiCoFe_2_O_4_@L-Silica@C-Niosome at different pH, and (**d**) curcumin from NiCoFe_2_O_4_@L-Silica@C-Niosome at different pH.

A release study of curcumin and letrozole from different samples was performed at different pH levels for 72 h. According to Figure 2a, the release rate of letrozole from the NiCoFe_2_O_4_@L-Silica formulation after 8 h at pH 7.4 was 28.7%; meanwhile, it reached 61.29% after 72 h. By covering NiCoFe_2_O_4_@L-Silica nanoparticles with a niosomal layer, the release rate of letrozole decreased, as only 48% of the loaded letrozole was released at a pH of 7.4. Similarly, the release profile of letrozole from NiCoFe_2_O_4_@L-Silica@L-Niosome formulations at different pH levels (7.4, 6.5, and 5.4) was analyzed. As shown in Figure 2b, by decreasing the pH, the release rate of letrozole increased, where the percentage of released drug at pH 7.4, 6.5, and 5.4 was 55%, 77%, and 89%, respectively.

Likewise, the release of curcumin and letrozole was investigated from NiCoFe_2_O_4_@L-Silica@C-Niosome formulations with three pH conditions (Figure 2c,d). The release rate of letrozole from formulations with pH 7.4, 6.5, and 5.4 was 51.28%, 62.19%, and 73.29%, respectively. Furthermore, the release rates of curcumin from the mentioned formulation at the same pH were 67.19%, 74.75%, and 84.3%, respectively. These outcomes showed that letrozole and curcumin release rates increased with the pH reduction and in acidic circumstance. Controlled release of the drugs from the niosome also increased with decreasing pH; thus, it can be predicted that nanocarriers are more likely to be released in an acidic environment of cancer cells, and targeted delivery leads to a reduction in the side effects of drugs in other environments as well. The release of letrozole and curcumin from the bilayer membrane of the niosome is related to the nature of these drugs and the composition and fluidity of the membrane. The interaction of the two studied drugs and the niosomal membrane surfactant is important in physiological pH. Studies have confirmed the biphasic release of anticancer drugs from the structure of the niosome, and it was found that the initial release is rapid and then enters a slow phase [43]. The pH-dependent release of drugs from the niosomal formulation is due to hydrolysis of the surfactant molecules under acidic pH and, therefore, disruption of the niosomal formulation [19]. In normal differentiated adult cells, the intracellular pH is generally ~7.2 and lower than the extracellular pH of ~7.4 [44]. Tumors have been demonstrated to exhibit acidic pH values ranging from 5.7 to 7.8 [45,46]. The acidic environment of tumor tissue is mainly derived from the glycolytic pathway and the catalytic action of carbonic anhydrase. The glycolytic pathway can produce lactic acid and the catalytic action can generate much carbonic acid [47,48]. Therefore, designing a nanocarrier that is sensitive to acidic pH and releases the loaded drug in such conditions is of interest for efficient cancer treatment.

### 2.3. Cytotoxicity Evaluation

The 3-(4,5-Dimethylthiazol-2-Yl)-2,5-Diphenyltetrazolium Bromide (MTT) assay is a common colorimetric approach to evaluate the preliminary cytotoxicity of both synthetic and natural materials against a wide range of cell lines [49]. To investigate the cytotoxicity of the as-developed nanocarrier in this study, we performed the MTT assay to calculate the IC50 value (i.e., concentration of drug that results in 50% cell viability) for different nanocarriers as well as the pure drug molecules against both cancerous breast cell lines (i.e., MDA-MB-231 and SK-BR-3) and a non-tumorigenic cell line (i.e., MCF-10A).

**Figure 3:**
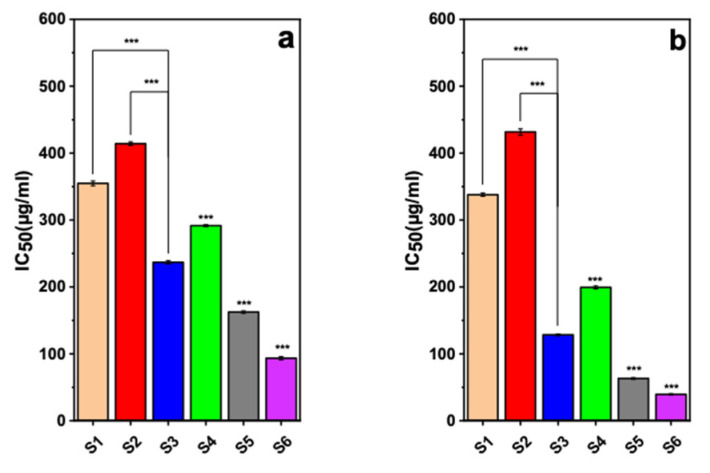
Investigation of cell viability for (**a**) MDA-MB-231 and (**b**) SK-BR-3 breast cancer cell lines using MTT assay. S1: letrozole; S2: curcumin; S3: mixture of letrozole and curcumin; S4: NiCoFe_2_O_4_@L-Silica@Niosome; S5: NiCoFe_2_O_4_@L-Silica@L-Niosome; S6: NiCoFe_2_O_4_@L-Silica@C-Niosome (*** *p* < 0.001).

The results suggest that mixing letrozole and curcumin led to a higher cytotoxicity against both studied cancerous cells, which can be attributed to the synergic effect between these two drugs [35,50]. This observation is in agreement with other research. For example, it has been reported that both curcumin-loaded poly lactic-co-glycolic acid (PLGA) nanoparticles and letrozole-loaded PLGA nanoparticles have been more powerful than free curcumin and free letrozole in inducing cancer cell apoptosis, and they improved cellular uptake and led to a growth in the rate of bioactivity in vitro and the premier bioavailability in vivo of curcumin and letrozole [33,51]. Additionally, we observed that loading these drugs into the nanocarriers resulted in higher cytotoxicity effects in both MDA-231 and SK-BR-3 breast cancer cell lines, as can be found in Figure 3. Indeed, the drug molecules have low solubility in the biological medium, and their internalization could be prevented by the cell membrane due to some unfavored interactions [52,53]. However, the nanocarriers can better penetrate the cells through the endocytosis process, and therefore, they could deliver a higher dosage of drugs into the breast cancer cells [54,55]. Figure 3 also indicates that the NiCoFe_2_O_4_@L-Silica@C-Niosome had considerably low IC50 values of about 50 and 100 µg/mL against MDA-321 and SK-BR-3 cells, respectively, allowing MDA-MB-231 SK-BR-3 co-delivery of a high dosage of letrozole and curcumin into the cancer cells and taking advantage of the synergic therapeutic effects between them [55,56]. For MCF-10A, as a non-tumorigenic cell line, the IC50 values for letrozole, curcumin, mixture of letrozole and curcumin, NiCoFe_2_O_4_@L-Silica@Niosome, NiCoFe_2_O_4_@L-Silica@L-Niosome, and NiCoFe_2_O_4_@L-Silica@C-Niosome were 587.27, 561.51, 401.3, 761.3, 3370.8, and 842.2 µg/mL, respectively. Table S3 shows a comparison for IC50 values reported for letrozole and curcumin loaded into different nanocarriers against different cell lines (MDA-MB-231 and SK-BR-3). As can be seen, the IC50 value for NiCoFe_2_O_4_@L-Silica@C-Niosome is one of the lowest reported values, indicating high cytotoxicity of the letrozole and curcumin after loading into this nanocarrier. This high cytotoxicity comes from the high loading capacity of the as-designed nanocarrier in this study as well as the drugs’ internalization and release into the cancer cell lines.

Furthermore, we investigated the possible cytotoxicity effect of the as-synthesized nanocarriers against the MCF-10A cell line as a non-tumorigenic cell line model (Figure S2). The results indicate that the encapsulation of letrozole and curcumin into the NiCoFe_2_O_4_@Silica@Niosome nanocarriers not only improved the biocompatibility of the drugs but also resulted in high biocompatibility for this nanocarrier with non-tumorigenic cell lines. The high biocompatibility with normal cells but high cytotoxicity against cancer cells can be attributed to two main reasons. First, the outer layer of the as-designed nanocarriers is made of niosomal structures, which are well known as highly biocompatible structures, and the second reason is due to the pH-dependent behavior of the niosomal formulation and the difference between the pH of healthy tissues and cancerous tissues [33,54].

### 2.4. Gene Expression Analysis

**Figure 4:**
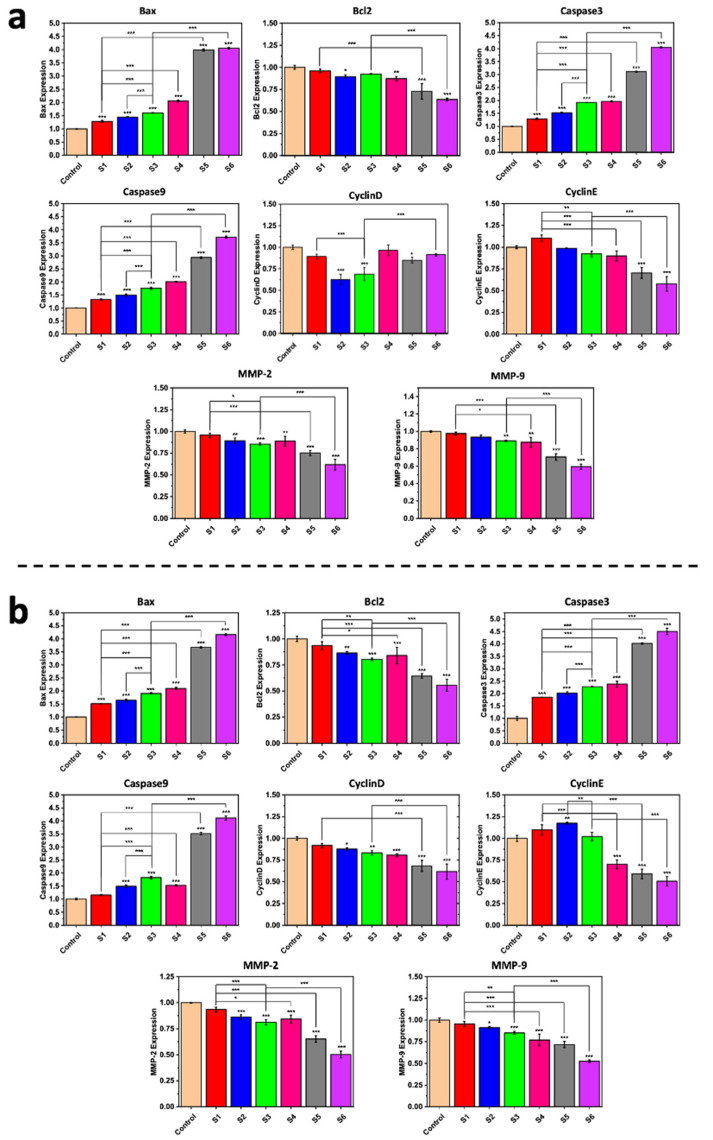
The expression of Bax, Bcl-2, caspase-3, caspase-9, MMP-2, MMP-9, cyclin D, and cyclin E in (**a**) MDA-MB-231 cell line and (**b**) SK-BR-3 cell line. S1: letrozole; S2: curcumin; S3: mixture of letrozole and curcumin; S4: NiCoFe_2_O_4_@L-Silica@Niosome; S5: NiCoFe_2_O_4_@L-Silica@L-Niosome; S6: NiCoFe_2_O_4_@L-Silica@C-Niosome; control: ß-actin (* *p* < 0.05, ** *p* < 0.01, *** *p* < 0.001). Treatments of range concentrations (IC50 concentration) of nano- and free drugs were applied to the cells for 48 h.

The emergence of real-time polymerase chain reaction (PCR) critically altered the field of measuring gene expression [57,58]. The use of gene expression profiling and the development of gene biomarkers/signatures for cancer allow for performing diagnosis, progression and aggressiveness analyses, prognosis, prediction of therapeutic treatment, and/or identification of patients who would benefit from therapeutic treatment and for better understanding the disease and its biology [59]. We assessed the caspase-3, caspase-9, Bcl-2, Bax, cyclin D, cyclin E, MMP-2, MMP-9, and ß-actin (internal control) gene expression levels in both MDA-MB-231 and SK-BR-3 cell lines by using real-time PCR analysis. As can be seen in Figure 4, during the research, we evaluated these genes’ expression after incubation of the breast cancer cells with different samples. These genes could be classified into two principal branches: pro-apoptotic and anti-apoptotic [60,61]. The outcomes indicate that the synergic effect between the two drug molecules was reflected in the higher regulation rate of various genes in both the MDA-MB-231 and SK-BR-3 cancerous breast cell lines compared to the use of each drug alone. Loading these drug molecules into nanoparticles further enhanced their beneficial role in diminishing genes’ expression level within cancerous breast cell lines. These findings are in agreement with those of numerous studies [62,63]. We observed overexpression of Bax when the cells were treated by NiCoFe_2_O_4_@L-Silica@C-Niosome, and a similar trend has been reported by Kordezangeneh et al. [64]. Similar trends also were observed for caspase-3 and caspase-9 genes.

Applying pure curcumin and letrozole to these cell lines also reduced the caspase-3 and caspase-9 expression levels. As proof, it is clear that the level of expression of caspase-3 and caspase-9 genes went down by using free drugs, and NiCoFe_2_O_4_@L-Silica@C-Niosome treatment increased these genes’ expression significantly (*** *p* < 0.001). Cur-NPs stimulated apoptosis of breast cancer cells via the caspase-3 and caspase-9 genes’ activation from approximately 4 to 3.55% in MDA-MB-231 and from approximately 4.5 to 4.25% in the SK-BR-3 cell line.

Dilnawaz et al. used curcumin-loaded magnetic nanoparticles (C-MNPs and Tf-CMNP) to direct K562 cells and derived a prompt reduction in the mitochondrial membrane that potentially led to cytochrome c release across the cytosol, followed by cleavage of caspase-3 and caspase-9 [19]. In the case of the Bcl2 gene, a NiCoFe_2_O_4_@L-Silica@C-Niosome sample influenced the gene expression rate toward negligible downregulation. The gene family of Bcl2 has an essential role in cancer cell apoptosis [14,65]. The alteration in the expression level of Bcl2 after the treatment of breast cancer cells with drug-loaded niosomes shows the prosperous delivery of the drug molecules into the SK-BR-3 and MDA-MB-231 cells and, consequently, the apoptosis of cells by downregulating the expression of Bcl2 gene. It was also reported that curcumin mainly repressed the Bcl2 expression in breast tumor cells [66]. Expression of MMP2 and MMP9 also considerably diminished due to use of NiCoFe_2_O_4_@L-Silica@C-Niosome in both investigated cell lines. MMP9 has a direct link to tumor growth, invasion, and metastasis, and estrogen has been shown to adjust the activity of MMP2 and MMP9 in breast cancer cells; thus, in estrogen-dependent breast cancer, we could confirm the invaluable role of nanocarriers that contain anti-breast cancer drugs. In addition, cyclin E and cyclin D expression levels were downregulated via NiCoFe_2_O_4_@L-Silica@C-Niosome. In the MDA-MB-231 and SK-BR-3 cell lines, we found that the expression rates for both cyclin genes were significantly reduced after treatment with NiCoFe_2_O_4_@L-Silica@C-Niosome. This significant reduction was observed in SKBR-3 cells from 1.0% for the control to 0.56 and 0.51% for cyclin E and cyclin D, respectively (*** *p* < 0.001), and a significant decrease from about 1.0% for the control to 0.9 and 0.55% for cyclin E and cyclin D, respectively (*** *p* < 0.001), was observed in the MDA-MB-231 cell line.

Breast cancer cell death is one of the important results that comes from successful anticancer drug delivery. Indeed, various metabolism rates in the cells lead to the death of cells and could be assessed by determination of the expression rate for different genes through anticancer drug delivery. We examined the ability of NiCoFe_2_O_4_@L-Silica@C-Niosome treatment to induce apoptosis in SK-BR-3 and MDA-MB-231 cells by gene expression. Based on this, it has been declared that the drug-loaded niosomes could advance the process of apoptosis in cancer cell lines via delivery of a high-level dose of drug molecules into cancer cells. This has different direct impacts on the expression levels of genes in the cells, and in turn, various ways for apoptosis could be activated (i.e., depending on drug and cell type), which could result in a high rate of apoptosis [16,67].

### 2.5. Flow Cytometry

**Figure 5:**
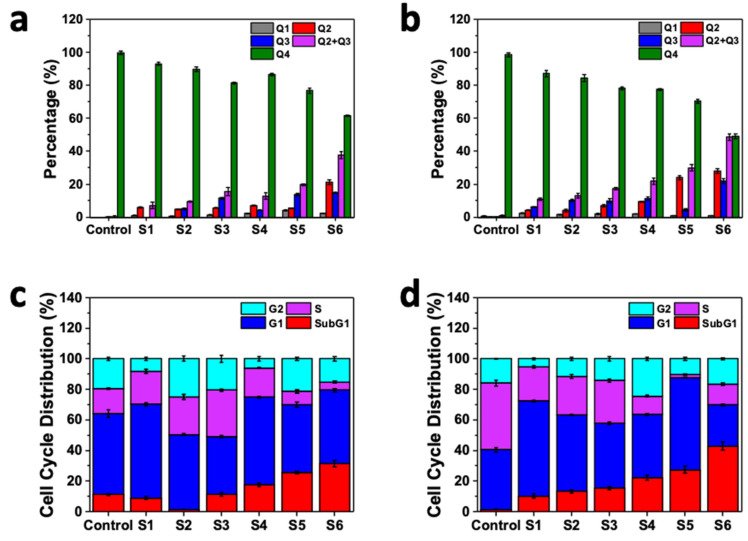
Analysis of the apoptosis rate in (**a**) MDA-MB-231 and (**b**) SK-BR-3 cancer cell lines after incubation with different samples. Q1 (necrotic cells), Q2 (late apoptosis), Q2 + Q3 (early apoptosis), and Q4 (alive cells). Treatments of range concentrations (IC50 concentration) of nano- and free drugs were applied to the cells for 48 h. Cell cycle analysis for (**c**) MDA-MB-231 and (**d**) SK-BR-3 cancer cell lines after incubation with different samples. S1: letrozole; S2: curcumin; S3: mixture of letrozole and curcumin; S4: NiCoFe_2_O_4_@L-Silica@Niosome; S5: NiCoFe_2_O_4_@L-Silica@L-Niosome; S6: NiCoFe_2_O_4_@L-Silica@C-Niosome. Control: untreated cells.

The efficacy of anticancer agents is usually determined through the cell survival and apoptosis. The apoptosis process is involved in the death of dividing cells and it is the necessary role of anticancer drugs [16,68,69]. In this study, the apoptosis rate of SK-BR-3 and MDA-MB-231 cells after treatment with different samples was measured. Moreover, we assessed untreated cell lines as control samples. Based on the results, there was a negligible change in Q1 (necrotic cells) after treatment with different drugs and drug-loaded nanocarriers. However, using letrozole and curcumin was more beneficial than using one drug alone for chemotherapy treatment of breast cancer. Loading effective drugs into nanocarriers enhances their benefits for the apoptosis of breast cancer cells. Therefore, applying both letrozole and curcumin via the designed magnetic niosomal nanocarriers altered the apoptosis rate of the breast cancer cell lines, as shown in Figure 5a,b.

In the SK-BR-3 cell line, the necrosis rate was reduced after treatment with NiCoFe_2_O_4_@L-Silica@C-Niosome. The amount of live cells also significantly increased from 0.02 to about 45% when we used a mixture of the two drugs, and this illustrates the enhanced cellular uptake when using nanocarriers, which have a positive impact on the synergic effect between letrozole and curcumin to eradicate breast cancer cell lines.

In the MDA-MB-231 cell line, the apoptosis rate steadily increased in all samples, while the maximum apoptosis rate was observed for the NiCoFe_2_O_4_@L-Silica@C-Niosome sample. In our previous research, we found that the pro-apoptotic and cytotoxicity impacts of curcumin niosomes were at a higher level than those of free curcumin when applied to cancer cell lines as a treatment, as niosomes increased cellular absorption and protected the bioactive cells [19]. When curcumin was combined with letrozole, their synergic effects were enhanced and were more beneficial in eradicating breast cancer cell lines (Table S4) [14,70]. The statistical comparison of the apoptosis rates of the studied samples is presented in Figure S3.

### 2.6. Cell Cycle Analysis

The cell cycle is the process by which eukaryotic cells duplicate and divide. This kind of cycle consists of two specific and distinct phases: interphase—consisting of G1 (Gap 1), S (synthesis), and G2 (Gap 2)—and the mitotic phase—M (mitosis). During interphase, the cell grows (G1), accumulates the energy necessary for duplication, replicates cellular DNA (S), and prepares to divide (G2). Each step in the cell cycle analysis is tightly adjusted, and checkpoints exist to distinguish possible DNA damage and allow it to be repaired prior to cell division. If the damage cannot be repaired, the cell becomes targeted for apoptosis [71,72]. In order to investigate the effect of our unique magnetic niosomal formulation containing letrozole and curcumin on the cell cycle process, the apoptosis rate, and the efficacy inhibition mechanism, we performed this analysis on MDA-MB-231 and SK-BR-3 breast cancer cell lines after 48-hour incubation and also on an untreated cell line as a control. As can be seen in Figure 5c,d, NiCoFe_2_O_4_@L-Silica@C-Niosome had the most significant SubG1 percentage among other samples, which is due to the enhanced cell uptake as well as the synergic effect between the two drugs loaded on the nanocarrier. Curcumin is able to disrupt the cell cycle through apoptosis in combination with other agents, leading to the activation of caspases as well as the downregulation of anti-apoptotic gene products Bcl-X [73,74,75]. Moreover, the S-phase of breast cancer cells that underwent curcumin niosome treatment showed significant cell cycle arrest, which shows a development of apoptosis [72]. These outcomes indicate that niosomes are a promising target in the delivery of drugs such as curcumin in breast cancer therapy. Similarly, letrozole is also able to inhibit the proliferation of cells by blocking cell progress during the cell cycle instead of having a direct cytotoxic impact [76]. Lisztwan et al. [77] showed that the inhibitory impact of letrozole is promoted in tumor cell proliferation via a mixture of prostaglandin E2 with growth factor I receptor [76]. According to the abovementioned evidence, we found that the remarkable impact of the mixture of letrozole and curcumin increased tumor growth inhibition, using just one tenth of the principal doses, and better prevented the growth of tumors. Finally, we observed that the SK-BR-3 cell line was affected more than the MDA-MB-231 cell line, as the SubG1 analysis showed that the apoptosis rate of SK-BR-3 cells was 41% compared to 34% in MDA-MB-231 cells. This is strong evidence showing that the SK-BR-3 apoptosis rate is higher than that of MDA-MB-231 cells in the presence of the samples. The cell cycle results in steps SubG1, G1, S, and G2 for all samples are presented in Table S5. The statistical comparison of the SubG1 % rate of the studied samples is presented in Figure S3.

### 2.7. Migration Analysis

Cell migration plays a principal role in the process and maintenance of organization of multicellular organisms, and aberrant cell migration is detected in numerous pathological diseases such as cancer [78,79]. Cancer cell migration is a critical process for distant metastases. Cell migration involves the movement of individual cells, cell sheets, or cell groups from one location to another [80]. Two significant types of migration include single cell migration and collective cell migration. Through collective cell migration, multiple cells perform an adjusted motion coordinated by cell–cell adhesion, collective cell polarization, coordination of cytoskeletal activity, and chemical and mechanical cues [81]. Using in vitro assays enables us to quantify the cell migratory capacity under controlled experimental conditions [80]. To evaluate the progression of breast cancer cells in this study, the breast cancer cells were treated with NiCoFe_2_O_4_@L-Silica@C-Niosome (i.e., sample S6), which showed the highest cytotoxicity against both studied breast cancer cell lines, and the percentage of cell migration was assessed by determining the number of cells moving through the microporous membrane (transwell migration assay) [82].

**Figure 6:**
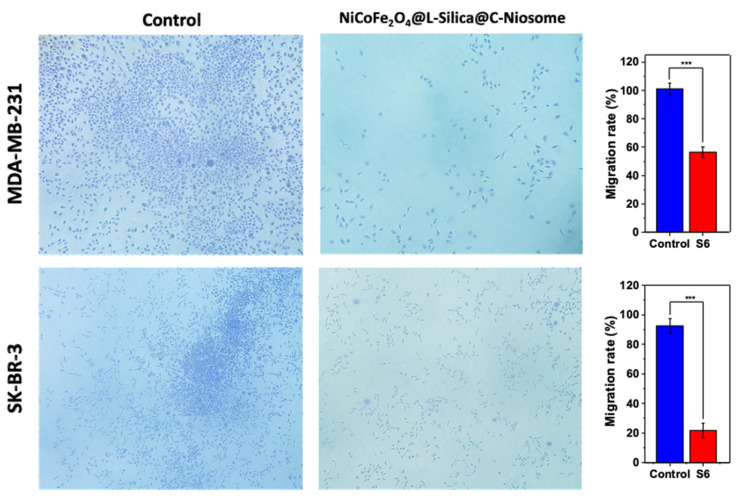
Migration rate in both cell lines after treatment with NiCoFe_2_O_4_@L-Silica@C-Niosome, presented as bar plots. Control: untreated cells. (*** *p* < 0.001). The treatment lasted 48 h for all tests at IC50 values.

As can be seen in Figure 6, the migration was impaired and inhibited significantly by 59 ± 3.54% in MDA-MB-231 cells after treatment with the magnetic niosomal formulation, whereas control cells—i.e., untreated cell lines—nearly doubled to 100 ± 4.24%. Similarly, in the SK-BR-3 cell line, NiCoFe_2_O_4_@L-Silica@C-Niosome treatment also resulted in a 25 ± 4.95% reduction in cells’ movement. Control cells showed a significant increase in their movement against NiCoFe_2_O_4_@L-Silica@C-Niosome, which was 96 ± 4.15%. In summary, the inhibitory effect of NiCoFe_2_O_4_@L-Silica@C-Niosome on cell migration was observed in both cell lines.

## 3. Materials and Method

### 3.1. Materials

Letrozole and curcumin were received as gifts from Tofigh-Darou Co., Tehran, Iran. Sorbitan monostearate (Span60), tween 60 (Polysorbate 60), cholesterol, sodium dodecyl sulfate (SDS), phosphate-buffered saline (PBS), dimethyl sulfoxide (DMSO), chloroform, ferric chloride, Nickel(II) chloride hexahydrate, cobalt chloride hexahydrate, sodium hydroxide, ethanol, tetraethyl orthosilicate, ammonia solution (25%), naproxen, methanol, Amicon (Ultra-15 Membrane, MWCO 30 kDa), and dialysis membrane (MWCO 12,000 Da) were purchased from Sigma Aldrich (St. Louis, MO, USA). MDA-MB-231, SK-BR-3, and MCF-10A cell lines were received from the Cell Bank of Pasture Institute (Tehran, Iran).

MTT (dimethylthiazol-2-yl-)-2,5, and penicillin/streptomycin (PS, 100 X) were purchased from Gibco (New York, NY, USA). Trypsin-EDTA, Trypan blue, Medium RPMI 1640, Dulbecco’s Modified Eagle Medium (DMEM), fetal bovine serum (FBS), and an Annexin V-FITC Flow cytometry kit were purchased from Invitrogen (California, CA, USA). RNA Extraction and cDNA Synthesis kits (Cat No. ER101-01 and AE301-02) were obtained from Transgene Biotech (Beijing, China). Milli-Q water (Millipore, Darmstadt, Germany) was used for making aqueous solutions. All other chemicals used were of analytical grade.

### 3.2. Synthesis of NiCoFe_2_O_4_ Nanoparticles

The NiCoFe_2_O_4_ nanoparticles were synthesized using a co-precipitation approach described in the literature [2].

### 3.3. Growth of Silica Shell on NiCoFe_2_O_4_ Nanoparticles and Loading Letrozole

The as-synthesized NiCoFe_2_O_4_ nanoparticles (500 mg) were dispersed in a mixture of various solvents, including 20 mL ethanol, 3 mL deionized water, and 1 mL ammonia solution (25%), in an ultrasonic bath for 20 min. Then, 0.7 mL of TEOS was added (dropwise) to the mixture. The product was separated by a magnet after 12 h of stirring under nitrogen atmosphere at room temperature. Finally, the NiCoFe_2_O_4_@Silica nanoparticles were washed with water and ethanol several times and dried at 25 °C. To load letrozole onto the NiCoFe_2_O_4_@Silica NPs, 0.04 g of nanocarrier was added to a solution of letrozole (20 mL, 1 mg/mL) and stirred in a dark place for 24 h (120 rpm). The suspensions were centrifuged at 14,000× *g* for 45 min at 4 °C. The letrozole loading efficacy was 61.32% in the NiCoFe_2_O_4_@L-Silica sample, calculated by a UV-Vis spectroscopic method.

### 3.4. Formation of Niosomal Layer on NiCoFe_2_O_4_ @L-Silica

First, 10 mg of the as-prepared NiCoFe_2_O_4_ @L-silica was dissolved in 5 mL methanol and sonicated for 1 min to disperse completely. Then, the mixture of curcumin (10 mg), span 80 (76.29 mg), DCP (2.44 mg), and cholesterol (34.41 mg) in 5 mL chloroform, was added to the letrozole-loaded NiCoFe_2_O_4_@Silica solution. The process for the formation of the niosomal layer was similar to our previous work [19].

### 3.5. Instrumentation

A Hitachi 295 FTIR spectrophotometer was used to obtain the FTIR spectra of different samples. The samples were mixed with KBr and the prepared discs were used for analysis of Fourier transform infrared (FTIR) spectroscopy.

The magnetic property of the nanoparticles was measured using a Gouy magnetic balance (FD-TX-FM-A, Dazhan Instrum. Inc., Nanking, China) and a vibrating sample magnetometer (VSM, BHV-55, Instrum. Inc. of Nanking University, Nanking, China). Surface area measurements were performed on a Micromeritics ASAP 2020 surface area and porosity analyzer (Quantachrome, Florida, FL, USA). The samples were out-gassed overnight (12 h) under nitrogen prior to adsorption measurement. Pore distributions and pore volume were calculated using the adsorption branch of the N_2_ isotherms based on the Brunauer–Emmett–Teller (BET) and Barrett–Joyner–Halenda (BJH) models. The specific surface area was calculated on the basis of the BET equation.

The size and morphology of the as-synthesized nanocarriers were investigated by several techniques. The average distribution sizes of niosomes were estimated using a dynamic light scattering (DLS) technique by Malvern Zeta Sizer (Malvern Instrument, Malvern, UK). Deionized water was used to dilute niosome formulations (1:100) to avoid multiple scattering phenomena, since it has a crucial role to decrease antiparticle interactions. Subsequently, the morphology of niosomes was investigated using transmission electron microscopy (TEM). The sample was imaged with TEM at 100 kV (Zeiss EM900 Transmission Electron Microscope, Oberkochen, Germany).

### 3.6. Encapsulation Efficiency

The encapsulation efficiency for each drug was calculated according to the protocol presented in our previous work [19]. The quantity of the loaded drug was estimated by measuring the absorption of each drug at its maximum absorption peak (240 nm for letrozole and 420 nm for curcumin) using UV–visible spectrophotometry. The calibration curve for each drug can be found in Figure S1. The encapsulation efficiency was measured by the following equation:

(1) $$encapsulation\;efficiency\left(EE\%\right)=\frac{initial\;drug\;added\left(\mathrm{mg}\right)-free\;drug\left(\mathrm{mg}\right)}{initial\;drug\;added\left(\mathrm{mg}\right)}\times 100$$

### 3.7. Release Study

In vitro release of the samples was accomplished using a dialysis tube in a PBS solution, following the procedure described in the literature [19].

### 3.8. Cytotoxicity of Cells (In Vitro Analysis)

A comparison of the cytotoxic impacts of nano-drugs with those of free drugs on cell viability was performed by an MTT assay for the MDA-MB-231, SK-BR-3, and MCF-10A cell lines, following the protocol described in our previous work [19]. During the MTT test, positive and negative controls were considered as follows: Positive control: untreated cells + MTT reagent + DMSO; Negative control: untreated cells + MTT + solubilizing buffer (without any samples, 10% SDS in 0.1 N HCL in our case); Blank: untreated cells+ MTT reagent + empty niosome.

### 3.9. Real-Time PCR Analysis

All nanocarrier samples as well as free drug molecules in the solution were introduced to MDA-MB-231 and SK-BR-3 cells at their IC50 concentrations and incubated for 2 days. Isolation of the whole RNA from cultured MDA-MB-231 and SK-BR-3 cells and synthesis of cDNA were performed by following the previously reported protocol [19].

### 3.10. Apoptosis Analysis (Flow Cytometry)

In MDA-MB-231 and SK-BR-3 cells, apoptosis activities were quantified via apoptosis assay using flow cytometry followed by cell treatment for 48 h, as mentioned in our previous work [19]. The apoptosis rates in the studied breast cancer cell lines after treatment with different samples are provided in Table S4. In this table, Q1, Q2, Q2 + Q3, and Q4 correspond to necrotic cells, late apoptosis, early apoptosis, and live cells, respectively. The alternative to apoptotic cell death is necrosis (Q1), which is considered to be a toxic process where the cell is a passive victim and follows an energy-independent type of death. Early-stage apoptosis (Q2 + Q3) is represented by alterations to, and ultimate loss of, the mitochondrial membrane potential. Identification of late-stage apoptosis (Q2) was performed by looking at the defragmentation of DNA. Early-stage apoptotic cells (Q2 + Q3) are Annexin-V-positive and PI-negative (Annexin V-FITC^+^/PI^−^), while late (end-stage) apoptotic cells (Q2) are Annexin V/PI-double-positive (Annexin V-FITC^+^/PI^+^). 

### 3.11. Cell Cycle

Cell proliferation evaluation was performed by utilizing propidium iodide (PI) staining. Detection of the cell cycle stage was performed by investigation of the DNA content, wherein the binding of PI to DNA is proportional to the DNA content. Cells were seeded in a complete medium in 6-well plates at a density of 1 × 10^6^ cells/well and incubated overnight. PBS was used for washing and plating the cells 3 times. Then, the cells underwent drug-loaded patch treatment for 48 h in a complete medium. After incubation, they were separated and fixed with 70% cold ethanol overnight at 4 °C and stained with 500 μL of PI solution in the dark for 20 min at room temperature. Finally, they were analyzed by flow cytometry. All above trials were repeated 3 times.

### 3.12. Migration Assay—Cell Migration Efficacy

Investigation of the anti-metastatic aspects of each formulation was performed by the “migration assay” in cell lines. The migration of MDA-MB-231 and SK-BR-3 cells was performed using 8 μm transwell chambers (Corning, New York, NY, USA). In this method, MDA-MB-231 and SK-BR-3 cells at a density of 5 × 10^4^ cells per well were placed in an upper chamber with no serum medium, while the lower chamber was filled with 500 μL of medium supplemented with 20% FBS. After 48 h incubation with NiCoFe_2_O_4_@L-Silica@C-Niosome, the cells migrating to the lower surface of the membrane were stained and counted. The percentage of migrated cells was examined by counting cells under a microscope (Olympus, Tokyo, Japan) in 3 randomly selected fields [37].

### 3.13. Statistical Analysis

Statistical analysis and curve fitting were carried out using OriginPro (2018) and GraphPad Prism software (version 8). All data were reported as mean ± SD (SD: standard deviation) from three independent experiments. One-way and two-way analyses of variance (ANOVAs) were used to measure the significance level (* *p* < 0.05, ** *p* < 0.01, *** *p* < 0.001) of differences between groups.

## 4. Conclusions

In summary, we have developed a magnetic niosomal nanocarrier using supermagnetic NiCoFe_2_O_4_ nanoparticles, followed by formation of a thin layer of silica and a niosomal structure. This unique approached allowed us to load two different drugs (i.e., letrozole and curcumin) on the as-developed nanocarrier and investigate the potential of using such a magnetic niosomal nanocarrier to fight breast cancer. The results showed that the as-developed nanocarriers were highly biocompatible with non-tumorigenic cell lines, while they possessed high cytotoxicity against the studied breast cancer cell lines (i.e., MDA-MB-231 and SK-BR-3). The high cytotoxicity was found to be due to the pH-dependent release behavior of the drug molecules from the nanocarriers as well as the synergic effect between the two drugs. This synergic effect was confirmed by several tests, such as gene expression level and cell cycle analysis. The results indicated that the co-delivery of letrozole and curcumin led to a higher apoptosis rate in the cancer cells, which was mainly due to regulation of the expression level of various genes inside the breast cancer cells. The cell migration investigation also revealed that co-delivery of letrozole and curcumin by such a magnetic niosomal formulation could slow down the migration level of breast cancer cells significantly, which prevents the spreading of cancer cells inside the body and, in turn, postpones the metastasis stage. All in all, the as-designed magnetic niosomal formulation showed great potential for use in effective chemotherapy treatment of breast cancer. The result of this study might open up new avenues to the future of nanomedicine and can help the scientific community to gain better insight into the design of smart nanocarriers for future cancer treatment.

## Figures and Tables

**Table 1:** Analysis of different samples by dynamic light scattering technique at different stages of development of the magnetic nanocarrier.

Average Hydrodynamic Diameter (nm)	PDI	SAMPLE
45.24	0.039	NiCoFe_2_O_4_
67.11	0.222	NiCoFe_2_O_4_@Silica
83.14	0.098	NiCoFe_2_O_4_@L-Silica
90.91	0.239	Bare Niosome
120.1	0.057	NiCoFe_2_O_4_@L-Silica@Niosome
122.5	0.137	NiCoFe_2_O_4_@L-Silica
138.7	0.236	NiCoFe_2_O_4_@L-Silica@C-Niosome
L-Silica: letrozole-loaded silica; C-Niosome: curcumin-loaded niosome		

## Data Availability

The data presented in this study are available upon request from the corresponding author. The data are not publicly available due to privacy restrictions.

## Supplementary Materials

The following are available online at https://www.mdpi.com/article/10.3390/ijms22157948/s1.
