# Supplementary material for: Super Magnetic Niosomal Nanocarrier as a New Approach for Treatment of Breast Cancer: A Case Study on SK-BR-3 and MDA-MB-231 Cell Lines
Source: Int J Mol Sci. 2021 Jul 26;22(15):7948. doi: 10.3390/ijms22157948 (PMC8347826; doi:10.3390/ijms22157948)
Supplement: Supplementary file 1 [file ijms-22-07948-s001.zip › ijms-1281245-supplementary.pdf]

Supporting Information
Supermagnetic niosomal nanocarrier as a new approach for treatment of breast cancer: A case study on SK-BR-3 and MDA-MB-231 cell lines

Elham Jamshidifar[a], Faten Eshrati Yeganeh[b, *], Mona Shayan[c], Mohammad Tavakkoli Yaraki[d,*], Mahsa Bourbour[e], Ali Moammeri[f], Iman Akbarzadeh[g], Hassan Noorbazargan[h], Nikoo Hossein-Khannazer [g, *]

[a] Department of Pharmaceutical Nanotechnology, Faculty of Pharmacy, Tehran University of Medical Sciences, Tehran, Iran.
[b] Department of Chemistry, Science and Research Branch, Islamic Azad University, Tehran, Iran.
[c] Department of Biology, Mashhad Branch, Islamic Azad University, Mashhad, Iran
[d] Research and Development Department, Nanofy Technologies Pte. Ltd., 049422, Singapore
[e] Department of Biotechnology, Alzahra University, Tehran, Iran.
[f] School of Chemical Engineering, College of Engineering, University of Tehran, Tehran, Iran.
[g] Gastroenterology and Liver Diseases Research Center, Research, Institute for Gastroenterology and Liver Diseases, Shahid Beheshti University of Medical Sciences, Tehran, Iran.
[h] Department of Biotechnology, School of Advanced Technologies in Medicine, Shahid Beheshti University of Medical Sciences, Tehran, Iran.

*Corresponding Authors:
Dr. F. Eshrati Yeganeh, E-mail: ffyeganeh@gmail.com
Dr. M. Tavakkoli Yaraki, E-mail: mty206@yahoo.com; mohammad@nanofy.co
Dr. N. Hossein-Khannazar, , E-mail: nikookhannazer@gmail.com

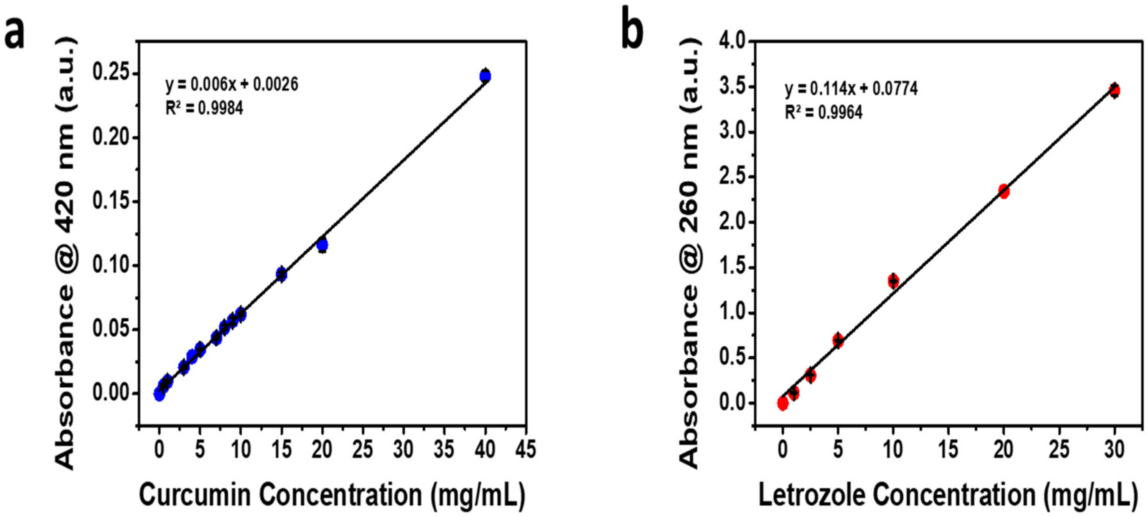

Figure S1. The calibration curve for absorbance of curcumin (a) and letrozole (b) versus their concentrations

**Table S1.** The sequence of primers used in real-time PCR analysis.	
**Gene**	**Sequence of Primers**
Cyclin D	Forward: 5'- CAGATCATCCGCAAACACGC-3′<br>Revers: 5'- AAGTTGTTGGGGCTCCTCAG-3'
b-actin	Forward: 5’- TCCTCCTGAGCGCAAGTAC-3’<br>Revers: 5’- CCTGCTTGCTGATCCACATCT-3’
Caspase 3	Forward: 5'- CATACTCCACAGCACCTGGTTA-3′<br>Revers: 5'- ACTCAAATTCTGTTGCCACCTT-3'
Caspase 9	Forward: 5’-CATATGATCGAGGACATCCAG-3<br>Revers: 5’-TTAGTTCGCAGAAACGAAGC-3’
Cyclin E	*Forward: 5'- CTCCAGGAAGAGGAAGGCAA-3′*<br>*Revers: 5'- TTGGGTAAACCCGGTCATCA-3'*
Bax	*Forward: 5'-CGGCAACTTCAACTGGGG-3′*<br>*Revers: 5'- TCCAGCCCAACAGCCG-3′*
Bcl2	*Forward: 5'- GGTGCCGGTTCAGGTACTCA-3′*<br>*Revers: 5'- TTGTGGCCTTCTTTGAGTTCG-3′*
P53	*Forward: 5'- CATCTACAAGCAGTCACAGCACAT-3′*<br>*Revers: 5'- CAACCTCAGGCGGCTCATAG-3′*

Table S2. Magnetic properties of $NiCoFe_2O_4$ and $NiCoFe_2O_4$@Silica nanoparticles by VSM

Sample	Saturation magnetization (Ms) (emu/g)	Remnant field (Mr) (emu/g)	Coercivity (Hc) (gauss)
NiCoFe2O4	17.58	5.55	805
NiCoFe2O4@Silica	11.71	4.06	1013

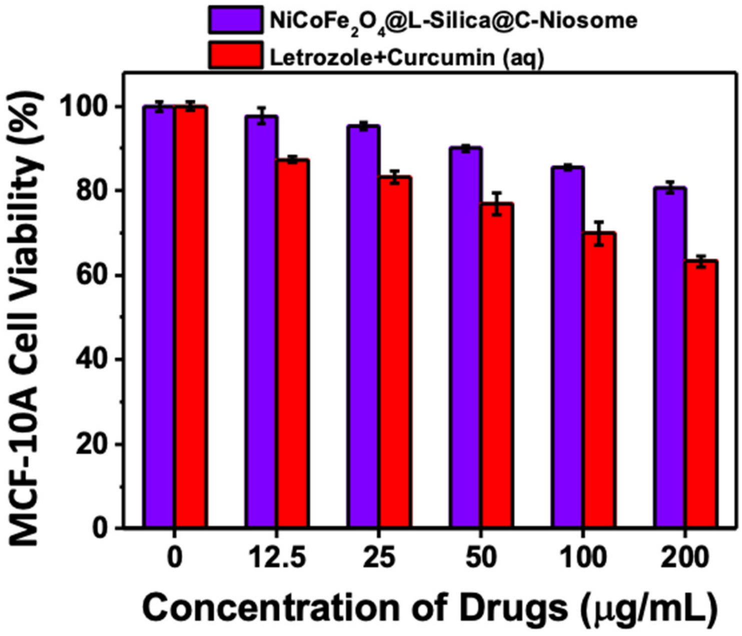

Figure S2. cytotoxicity effect of the as-synthesized nanocarriers against MCF-10A cell line

Table S3. A comparison for change in the IC50 values for different drugs before/after loading to different nanocarriers against and after incubation with different cell lines					
Drug	Cell line	Time (hr)	Form	IC50	**Ref**
Curcumin	SK-BR-3	72	Free drug<br>Loaded on niosomal formulation	127.39 µg/ml<br>69.77 µg/ml	**[5]**
Curcumin	MDA-MB-231	72	Free drug<br>Loaded on niosomal formulation	175.91 µg/ml<br>83.68 µg/ml	**[5]**
Melittin	SK-BR-3	48	Free drug<br>Nano	87.87 µg/ml<br>47.65 µg/ml	**[6]**
		72	Free drug<br>Loaded on niosomal formulation	50.56 µg/ml<br>31.05 µg/ml	
Letrozole<br>Curcumin	MDA-MB-231	48	Free Letrozole<br>Free Curcumin<br>$NiCoFe_2O_4$@L-Silica@C-Niosome	354.81 µg/ml<br>414.15 µg/ml<br>93.47 µg/ml	**This Study**
Letrozole<br>Curcumin	SK-BR-3	48	Free Letrozole<br>Free Curcumin<br>$NiCoFe_2O_4$@L-Silica@C-Niosome	337.82 µg/ml<br>431.62 µg/ml<br>39.48 µg/ml	**This Study**

Table S4. apoptotic rate in presence of samples.

MDA-MB-231

	Q1		Q2		Q3		Q2+Q3		Q4	
	Mean	SD	Mean	SD	Mean	SD	Mean	SD	Mean	SD
**Control**	0	0	0.012	0	0.47	0.005	0.34	0.40	99.9	1.1
**S1**	1.06	0.1	5.81	0.35	0	0	7.10	1.82	93.1	0.85
**S2**	0.46	0.08	4.7	0.21	5.05	0.42	9.43	0.46	89.79	1.34
**S3**	1.39	0.19	5.69	0.29	11.5	0.57	15.54	2.33	81.4	0.58
**S4**	2.22	0.24	7.05	0.08	4.28	0.21	12.80	2.07	86.5	0.72
**S5**	3.98	0.35	5.4	0.12	13.8	0.66	19.65	0.64	76.8	1.58
**S6**	2.37	0.07	21.3	1.34	14.8	0.18	37.70	2.26	61.5	0.37

SKBR3

	Q1		Q2		Q3		Q2+Q3		Q4	
	Mean	SD	Mean	SD	Mean	SD	Mean	SD	Mean	SD
**Control**	0.684	0.05	0.289	0.041	0.289	0.01	0.81	0.32	98.7	1.24
**S1**	2.45	0.19	4.28	0.22	6.09	0.28	10.94	0.80	87.2	1.92
**S2**	1.44	0.031	3.95	0.74	10.2	0.79	13.13	1.45	84.4	2.21
**S3**	1.96	0.25	6.96	0.66	9.87	1.21	17.32	0.69	78.21	0.79
**S4**	1.94	0.07	9.29	0.19	11.2	0.97	21.85	1.92	77.5	0.68
**S5**	0.838	0.1	24.1	1.25	4.56	0.74	29.96	1.83	70.5	1.25
**S6**	0.963	0.02	28.1	1.34	21.9	1.34	48.63	1.94	49	1.47

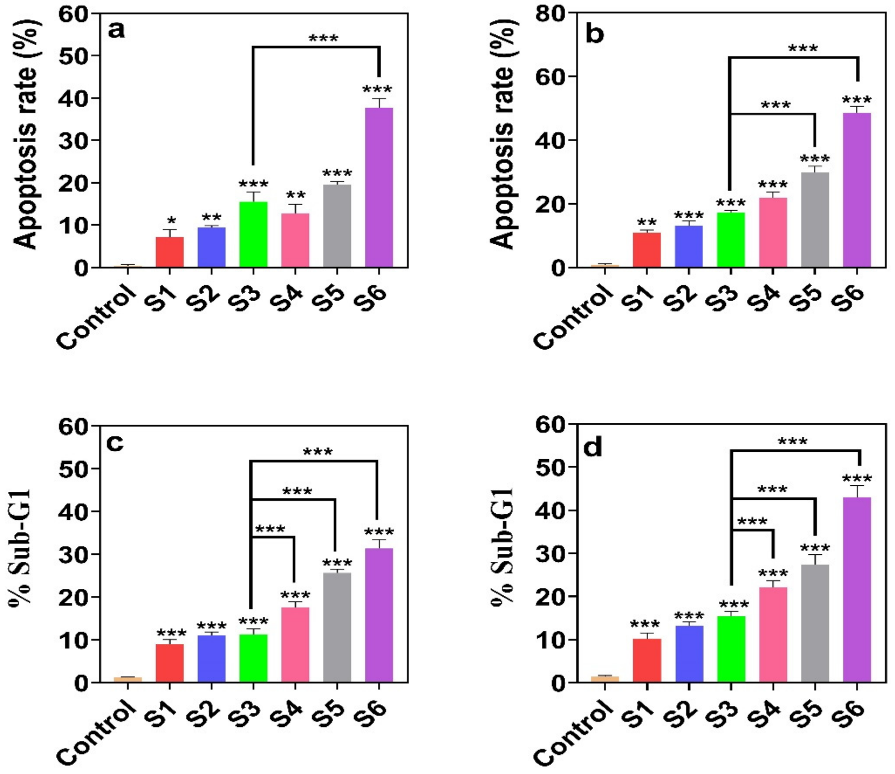

Figure S3. Statistical analysis for the apoptosis rate and Sub-G1% (* p<0.05, ** p<0.01, *** p<0.001) in different cell lines after treatment with different samples. **a** and **c**) MDA-MB-231 cell line, and **b** and **d**) SK-BR-3 cell line.

Table S5. Cell cycle rate of samples

	SubG1		G1		S		G2	
**MDA-MB-231**								
	Mean	SD	Mean	SD	Mean	SD	Mean	SD
**Control**	1.2	0.12	49.24	0.67	24.46	1.68	25.1	1.68
**S1**	8.98	1.1	61.34	1.14	21.46	1.22	8.22	0.94
**S2**	11.11	0.78	53.07	2.57	16.45	0.39	19.37	1.1
**S3**	11.3	1.35	37.71	0.84	30.5	0.73	20.49	2.42
**S4**	17.56	1.42	57.3	0.57	19.2	0.29	5.94	1.33
**S5**	25.67	0.91	44.37	1.55	8.67	1.1	21.29	1.21
**S6**	31.4	2.13	48.13	1.02	5.37	0.58	15.1	1.48
**SKBR3**								
	SubG1		G1		S		G2	
	Mean	SD	Mean	SD	Mean	SD	Mean	SD
**Control**	1.4	0.28	39.21	1.44	43.64	1.88	15.75	0.3
**S1**	10.25	1.34	62.21	0.67	22.35	0.92	5.19	0.75
**S2**	13.24	0.92	50.1	0.33	25.09	1.34	11.57	1.25
**S3**	15.42	1.1	42.33	0.88	28.04	1.12	14.21	1.34
**S4**	22.19	1.54	41.37	0.49	12.12	0.64	24.32	0.92
**S5**	27.44	2.33	60.24	0.23	2.19	0.42	10.13	1.13
**S6**	42.94	2.75	26.95	0.68	13.54	0.92	16.57	0.68
